# Transcriptome-Guided Drug Repurposing for Aggressive SCCs

**DOI:** 10.3390/ijms23021007

**Published:** 2022-01-17

**Authors:** Roland Zauner, Monika Wimmer, Sonja Dorfer, Michael Ablinger, Ulrich Koller, Josefina Piñón Hofbauer, Christina Guttmann-Gruber, Johann W. Bauer, Verena Wally

**Affiliations:** 1 EB House Austria, Research Program for Molecular Therapy of Genodermatoses, Department of Dermatology and Allergology, University Hospital of the Paracelsus Medical University Salzburg, 5020 Salzburg, Austria; mo.wimmer@crcs.at (M.W.); so.dorfer@crcs.at (S.D.); m.ablinger@salk.at (M.A.); u.koller@salk.at (U.K.); j.d.pinon@salk.at (J.P.H.); c.gruber@salk.at (C.G.-G.); joh.bauer@salk.at (J.W.B.); v.wally@salk.at (V.W.); 2 Department of Dermatology and Allergology, University Hospital of the Paracelsus Medical University, 5020 Salzburg, Austria

**Keywords:** epidermolysis bullosa, organ transplant recipients, squamous cell carcinoma, drug repurposing

## Abstract

Despite a significant rise in the incidence of cutaneous squamous cell carcinoma (SCC) in recent years, most SCCs are well treatable. However, against the background of pre-existing risk factors such as immunosuppression upon organ transplantation, or conditions such as recessive dystrophic epidermolysis bullosa (RDEB), SCCs arise more frequently and follow a particularly aggressive course. Notably, such SCC types display molecular similarities, despite their differing etiologies. We leveraged the similarities in transcriptomes between tumors from organ transplant recipients and RDEB-patients, augmented with data from more common head and neck (HN)-SCCs, to identify drugs that can be repurposed to treat these SCCs. The in silico approach used is based on the assumption that SCC-derived transcriptome profiles reflect critical tumor pathways that, if reversed towards healthy tissue, will attenuate the malignant phenotype. We determined tumor-specific signatures based on differentially expressed genes, which were then used to mine drug-perturbation data. By leveraging recent efforts in the systematic profiling and cataloguing of thousands of small molecule compounds, we identified drugs including selumetinib that specifically target key molecules within the MEK signaling cascade, representing candidates with the potential to be effective in the treatment of these rare and aggressive SCCs.

## 1. Introduction

While cutaneous squamous cell carcinomas (SCC) arising in the general population are well treatable, in some patient cohorts they arise more frequently, are particularly aggressive, and are associated with poor prognosis. In organ transplant recipients (OTR), long-term immunosuppression is associated with a 65 to 250-fold increase in the risk of developing cutaneous SCCs, with the frequency recurrence and metastasis reflecting the aggressiveness of these tumors [1,2,3]. In these patients, decreased immune-mediated tumor surveillance and an increased susceptibility to infections, including those caused by exposure to oncogenic viruses, are likely direct consequences of immunosuppression. Indeed, 90% of cutaneous SCCs arising in OTR patients are human papillomavirus (HPV)-positive, compared with only 40% in immunocompetent individuals [4]. Additionally, immunosuppressive agents themselves can contribute to the initiation of skin cancers, by sensitizing cells to ultraviolet (UV)-radiation, and consequently to mutation, e.g., in the tumor-suppressor gene Tumor Protein 53 (TP53) [4].

Similar to OTR patients, individuals suffering from the rare genodermatosis recessive dystrophic epidermolysis bullosa (RDEB) develop particularly aggressive cutaneous SCCs, which is the leading cause of early death in these patients [5]. While the etiology of RDEB-SCCs is completely different to those arising in OTRs, and is attributed to the functional loss of the extracellular matrix protein type VII collagen (C7) due to congenital mutation, molecular analyses have uncovered mechanistic and phenotypic similarities between these two tumor entities. First, transforming growth factor (TGF)-ß, a key player in tumor development and progression that has been associated with epithelial-to-mesenchymal transition (EMT), was shown to be increased in both OTR- and RDEB-SCCs. Although TGF-ß is constitutively overexpressed in RDEB skin and tumors as a consequence to the loss of C7 [6,7], in OTRs TGF-ß up-regulation is triggered by commonly used immunosuppressants such as cyclosporine [8,9]. Second, and potentially associated with TGF-ß signaling, a subset of tumor cells in the tissue from both OTRs and RDEB patients present with an EMT-like phenotype (i.e., cells of a typical spindle-like shape). These risk factors are highly uncommon in cutaneous SCCs from otherwise healthy immunocompetent individuals; however, in the rare cases of developing metastatic disease (2–5%), traits of EMT could already be observed at the level of actinic keratoses and are discussed as prognostic markers for an aggressive behavior of these SCCs. Overall, high tumor diameters and perineural involvement are the key risk factors for metastasis of UV-induced cutaneous SCCs [4,10,11,12,13]. 

Furthermore, microbial triggers and responses to such, appear to be a factor shared by both tumor entities. While HPV infection, a major risk factor in OTR-SCCs, is uncommon in RDEB [14], the latter is associated with pathogenic bacterial infection [15]. Analysis of the mutational landscape of RDEB-SCC has highlighted the apolipoprotein B mRNA editing enzyme, and its catalytic polypeptide-like (APOBEC)-mediated cell-endogenous mutagenic processes as a critical driver of tumor development [16]. APOBEC cysteine deaminases are known components of host innate antimicrobial defenses that are also induced against HPV. As such, HPV-positive head and neck (HN)-SCC [17], also feature a predominant APOBEC mutational signature [16]. Indeed, significant parallels between HN- and RDEB-SCCs are observed at both the transcriptome [18] and miRNome level (data not shown), in addition to a high degree of resemblance between RDEB-SCC and OTR-SCC [19].

Thus, despite diverging etiologies, OTR-, RDEB-, and HN-SCCs feature distinct similarities, both at the genomic and transcriptomic level, as well as in clinical behavior, which distinguishes them from UV-induced SCCs in otherwise healthy individuals. Since OTR- and RDEB-tumors are relatively rare, which severely restricts the search for new therapy options, the fact that they share specific molecular patterns provides an opportunity to leverage the similarities in their gene expression signatures and match them with abundant data available for more prominent HN-SCCs. While mindful of the differences between these tumors (e.g., loss of C7 in RDEB, immunosuppression in OTR, difference in epithelial tissue type, in particular involvement of mucosa in HN-SCCs), we aimed here to perform transcriptome-guided drug screening, in order to identify drugs that target shared, critical cancer-related pathways, using computational methods for mining publicly accessible data sets for drug repositioning. 

Specifically, in this study we employed signature reversion, a method that relies on the assumption that a drug is able to reverse a disease-associated gene expression profile, thereby modulating the disease phenotype itself [20]. We were able to demonstrate a common activation of distinct cancer-associated signaling cascades based on gene expression analysis and to identify a set of compounds, which could be considered in a repurposing strategy to address the urgent need of additional treatment options in these rare cancers. 

## 2. Results

### 2.1. Gene Expression Overlaps between HN-, OTR-, and RDEB-SCC

**Figure 1:**
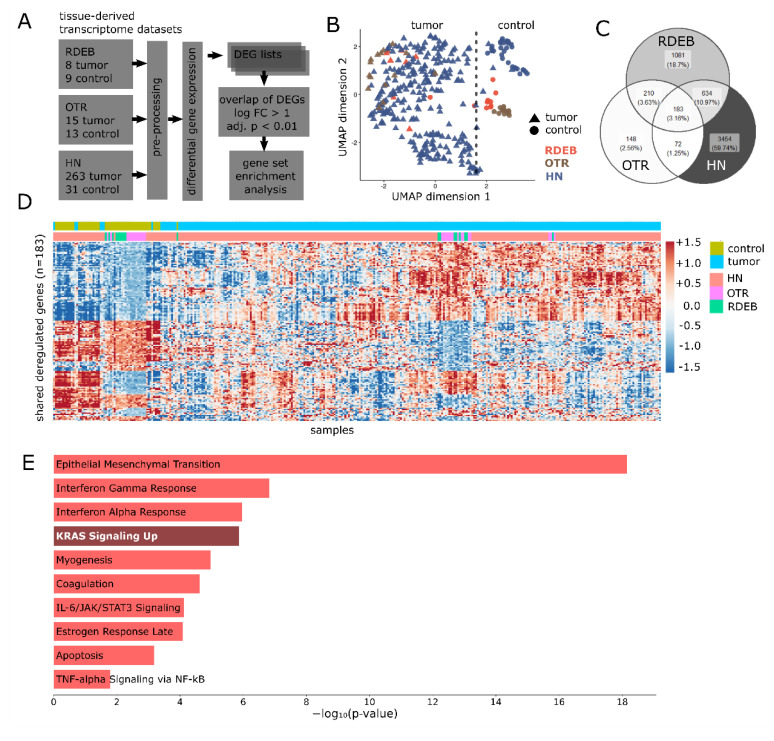
Analysis and evaluation of transcriptome similarities between organ transplant recipient (OTR)-, recessive dystrophic epidermolysis bullosa (RDEB)-, head and neck (HN)- squamous cell carcinoma (SCC). (**A**) Scheme of data processing workflow. (**B**) Uniform Manifold Approximation and Projection (UMAP) plot illustrates the distinct separation of tumor and control samples (dashed line) from all three data sets. All genes expressed in the three data sets were considered for dimension reduction. Gene expression data was z-transformed across samples within respective data sets. (**C**) Overlap of differentially expressed genes (DEGs) (min. 2-fold, adjusted-*p* < 0.01) between tissue data sets. (**D**) Heat map of 183 shared DEGs between all three data sets. Gene expression data were z-transformed. Columns: samples, Rows: DEGs. Samples and genes were organized by hierarchical clustering. (**E**) Plots show the Molecular Signature Database (MSigDB) hallmark gene sets significantly (adjusted *p* < 0.05) enriched in shared DEGs.

First, we were interested in extracting common gene expression patterns between RDEB-, OTR- and HN-SCC tissues (Figure 1A). Therefore, transcriptome datasets representative of all three tumor entities and associated non-tumor controls were retrieved from publicly available repositories (Table 1).

An unsupervised profiling of genes detected in all three tumor sets was conducted to assess similarities in gene expression. A UMAP plot illustrated a transcriptome-driven separation of samples according to their disease state (tumor vs. control-tissue), rather than their tumor type, further supporting our assumption of similarities between the three tumor sets (Figure 1B).

In order to derive matching deregulated gene patterns, we applied linear models (Limma) for normalized OTR (GSE32628) beadchip data, and the DESeq2 analysis pipeline for HN curated TCGA [22], as well as RDEB (GSE111582) RNA-seq data, obtaining a list of differentially expressed genes (DEGs) for each individual data set. Next, these DEG lists were compared to identify genes commonly deregulated across all data sets. In total, we identified 183 significantly deregulated genes (min. 2-fold, adjusted *p*-value < 0.01) common to all three cancer types, representing 8.6% of all deregulated genes in RDEB, 29.9% in OTR, and 4.2% in HN-SCCs (Figure 1C). A heatmap representing the 183 shared DEGs demonstrated a common gene expression pattern for RDEB- and OTR-SCC, as well as for a subset of HN-SCC within distinct tumor- and control-tissue clusters (Figure 1C). Thus, the integration of all three tumor data sets supported our hypothesis that the transcriptome profiles of OTR-, RDEB-, and HN-SCCs comprise shared, critical tumor-pathways. Moreover, functional analysis suggested a significant (adjusted *p*-value < 0.05) enrichment of the MSigDB hallmark gene sets representing major cellular programs known to be frequently hijacked by tumor cells, such as EMT or KRAS signaling (Figure 1E). Taken together, transcriptome profiles of OTR-, RDEB-, and HN-SCCs reflect common malignant cellular programs and substantiate our rationale that drugs potentially efficacious in all three tumor entities can be identified based on available transcriptome data.

### 2.2. In Silico Identification of Drugs for the Treatment of SCCs 

**Figure 2:**
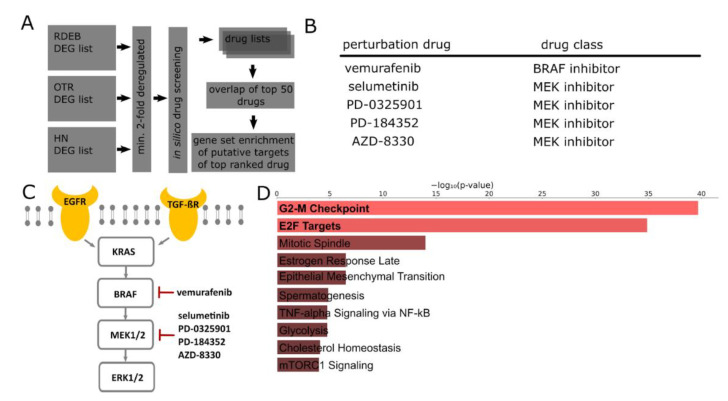
Drug candidate nomination. (**A**) Schematic workflow of the employed computational drug screening approach. (**B**) L1000CDS2 search engine-derived drug catalogues with common drugs targeting the MEK signaling cascade. (**C**) Schematic of the MEK signaling cascade and target molecules of selected perturbation drugs based on results of our in silico screening approach. (**D**) Barplot shows MSigDB hallmark gene sets significantly enriched in, e.g., up-regulated RDEB DEGs, which are putative targets of vemurafenib reported by the L1000 characteristic direction signature (CDS)2 drug screening algorithm.

The proposed computational drug screening approach is based on data and algorithms developed within the Library of Integrated Network-Based Cellular Signatures (LINCS) program [20]. This program was established to further enhance the understanding of cellular pathway responses to perturbation by small molecules (perturbome), capitalizing on publicly released omics data generated in the course of decades of therapeutic development (www.lincsproject.org, accessed 26 November 2021 [20]). To identify compounds able to reverse SCC-related cellular programs and potentially modulate a disease course, we screened publicly accessible drug perturbation datasets, specifically the L1000 catalogue, which contains over 33,000 chemically perturbed gene expression profiles based on more than 3900 small molecules. DEGs more than 2-fold up-/down-regulated in tumors vs. controls were used in a L1000CDS2 database query employing the reverse gene set mode. Separate queries were conducted for each tumor entity. The algorithm developed by the Ma’Ayan lab compared the submitted tumor gene expression signatures to differentially expressed genes computed from LINCS L1000 perturbome and returned descriptive information on 50 drug candidates that can potentially reverse the input signature for each tumor entity (Figure 2A) [23]. Drug candidates that appeared in all three screens were selected and reported with their respective mechanisms of action.

Of note, the most prominent top-ranked drug candidates were associated with a drug class targeting the MAPK/ERK-pathway (Figure 2B), which is in line with results from our previous gene set enrichment analysis (Figure 1E). This well-characterized kinase cascade is frequently triggered by oncogenic mutations (mostly RAS or RAF) or by growth factor signaling (e.g., EGFR), and its prominent role in many different types of cancers is reflected by the number of mitogen-activated protein kinase (MAPKK, aka MEK)-inhibitors currently under clinical investigation [24]. 

Within the MEK signaling cascade, four out of the five listed drugs directly target MEK, while one, vemurafenib, is a BRAF inhibitor (Figure 2C). An enrichment analysis performed on putative drug targets derived from the computational perturbation data screening, which were up-regulated in, e.g., RDEB-SCC, suggests a modulatory effect of vemurafenib on cell-cycle-related processes, and thereby further supports the meaningful applicability of our suggested approach (Figure 2D). 

### 2.3. Transcriptome Profiles Highlight Activation of MAPK Signaling 

**Figure 3:**
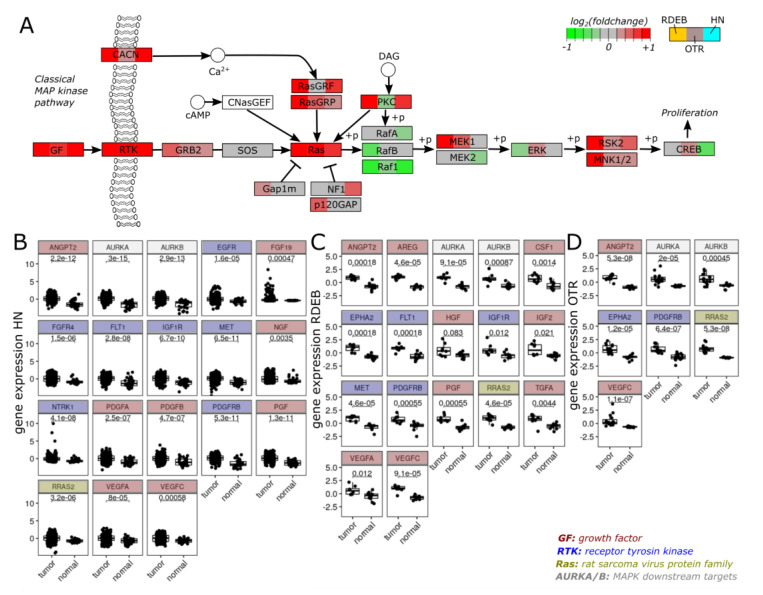
MAPK signaling activity. (**A**) Gene expression patterns of major classical MAP kinase pathway components (derived from KEGG pathway hsa04010). Node colors (green: down-regulated, red: up-regulated in tumor) illustrate relative gene expression levels (log2 foldchange of tumor vs. normal) in three sub-compartments (left box: RDEB, center box: OTR, right box: HN). In the case where the nodes represent multiple genes, e.g., GFs, the gene with the highest foldchange is visualized for the corresponding tumor entity. (**B**–**D**) Boxplots show gene expression of MAPK pathway members in (**B**) HN-, (**C**) RDEB- and (**D**) OTR-data sets belonging to functional class GF, RTK, RAS homologues, or are considered as downstream targets of MAPK signaling. Normalized counts (RDEB, HN) or intensity signals (OTR) were log2 and z-transformed to present the data on a common scale. Non-parametric, unpaired Wilcox test was applied to test significance.

As our in silico drug screening suggested compounds that were targeting critical players of the MAPK signaling, we were curious about an increase in the MAP kinase pathway activity supported by transcriptome data. Therefore, we assessed the overexpression of growth factors (GF), receptor tyrosine kinases (RTK) and RAS homologues associated with the Kyoto Encyclopedia of Genes and Genomes (KEGG)-derived classical MAPK signaling network (Figure 1A). Various extracellular signals able to initiate the signaling cascade as well as signaling receptors and mediators showed abundant expression in tumors, potentially contributing to MAPK activity. Stimuli, which appeared to be significantly up-regulated (min. 1.5-fold, adjusted *p*-value < 0.05) in all three tumors included angiopoietin 2 (ANGPT2) and vascular endothelial growth factor C (VEGFC), factors shown to act in concert to facilitate permissive angiogenic events [25,26]. Furthermore, an elevated expression of platelet-derived growth factor receptor ß (PDGFRB) was observed across all three tumors, which has been reported to promote distant metastasis in cancer stem cells of advanced skin carcinoma [27]. A common feature of all three tumors on the signal mediation level was the high level of Ras-related protein 2 (RRAS2) expression, which is in line with a study of malignant skin cancer demonstrating increased expression of RRAS2 in highly aggressive skin tumors [28]. Overall, several members of the MAPK pathway, including extracellular triggers, showed increased expression in malignant tissue and thus indicate aberrant MAPK signaling activity. This is further corroborated by the observed significant up-regulation of the mitotic serine/threonine kinase aurora kinase A/B (AURKA, AURKB) expression (Figure 3B–D), both being downstream targets of MAPK that contribute to the regulation of cell cycle progression [29]. 

## 3. Discussion

Drug repurposing, the discovery of novel applications for approved and investigational drugs, offers a cost-effective strategy for the treatment of human malignancies, facilitating rapid clinical translation [30]. While rare tumor types, such as RDEB- or OTR-SCCs, would greatly benefit from new therapeutic treatment options, research in this area is often challenging due to the scarcity of patients and sample material [31]. To overcome these limitations, we leveraged similarities between RDEB- and OTR-SCC and augmented our data set with the more common HN-SCC [16]. In the course of this study, we were able to substantiate the presence of overlapping gene expression patterns and could demonstrate consistent enrichment of pathways associated with malignancies across all three tumor types [10,32]. 

Our results specifically highlight the MAPK/ERK-signaling cascade to be commonly de-regulated in all datasets used in our study. Accordingly, drugs that target distinct players within this cascade were proposed by the L1000CDS2 search engine, with most targeting MEK directly, or upstream BRAF. Of note, BRAF inhibitors are commonly used in, e.g., melanoma treatment. However, in that context, hyperproliferative skin conditions due to paradoxical MAPK activation were a frequently seen side effect of treatment [33]. Interestingly, Escuin-Ordinas et al. showed that in two murine wound models, vemurafenib accelerated cutaneous wound healing, without increasing the incidence of SCCs [34]. This is of particular interest for the RDEB patient population, as these patients suffer from extreme skin fragility and chronic wounds. Such chronic wounds are the primary site of emergence of SCCs [35]. Therefore, it is essential that any tumor treatment applied in this patient population shows efficacy against the tumor, without inhibiting wound healing processes [36]. Furthermore, upon the co-administration of vemurafenib with the MEK inhibitor trametinib, hyperproliferative skin changes, as observed during melanoma treatment, improved significantly [37]. This could also be considered for HN- and OTR-SCC patient populations, although the negative effects associated with trametinib monotherapy on wound healing, as well as other reported cutaneous side effects (e.g., rash, increased skin infection by *Staphylococcus aureus*) [34,37], need to be stringently evaluated in EB patients. Taken together, there is rationale to investigate the use of vemurafenib alone, or the use of MEK-inhibitors locally only for SCC treatment in these patient cohorts. 

MEK inhibition is a treatment already clinically approved and frequently used for the treatment of melanoma, and clinical trials investigating the efficacy of MEK inhibitors for non-small cell lung cancer, colon cancer and thyroid cancer are currently ongoing (e.g., NCT03085056, NCT03704688). For RDEB, MEK inhibition has so far only been described in the context of the development of a PLK1-targeting therapy. Rigosertib, an inhibitor of polo-like kinase 1 (PLK1), was identified as a promising drug candidate for the treatment of RDEB-SCC, and was shown to induce G2-M cell cycle arrest and apoptosis in RDEB-SCC cells, as well as inhibiting tumor growth in xenograft studies in vivo. In this context, we also identified PLK1 as part of a protein interaction network derived from putative vemurafenib targets (data not shown), which is consistent with studies showing that rigosertib exerts a minor effect on MEK by competing with RAS/RAF interaction; however, this showed only a minor effect [38,39]. Currently, rigosertib is under clinical investigation in a phase I clinical trial for RDEB (NCT04177498).

For HN-SCCs, approved therapies include platinum-based chemotherapy regimens, checkpoint-inhibitors such as pembrolizumab, and the EGFR-targeting antibody cetuximab, as well as combinatorial therapies [40]. In addition, the thymidylate synthase inhibitor methotrexate, which is also listed as an approved drug for the treatment of HN-SCC, was recently identified in an in silico approach similar to the method describe here, as a potential candidate for the treatment of RDEB wounds [41]. While not currently approved for the therapy of HN-SCC, MEK-inhibition was shown to enhance the efficacy in combinatorial approaches, e.g., with PD-L- targeting antibodies or serine-threonine kinase CK2 inhibitors in preclinical investigations [42]. 

A more detailed analysis of the MAPK signaling cascade illustrated an increase in signaling activity in tumors based on the over-expression of growth factors, receptors and signaling mediators. In addition to supporting our assumption that the proposed compounds targeting the MAPK/ERK-pathway have potential use in restricting unchecked tumor growth, the gene expression data of MAPK signaling components provide further insights into molecular pathomechanisms. Our analysis highlighted an integration of various factors to promote angiogenesis across all tumor entities. Despite several similarities, we also found evidence for tumor-specific signaling programs. RDEB tumor profiles indicated altered hepatocyte growth factor (HGF)/hepatocyte growth factor receptor (HGFR/MET) and insulin-like growth factor II (IGF2)/insulin-like growth factor I receptor (IGF1R) dynamics, whereas HN-SCC appeared to take advantage of mitogenic fibroblast growth factor-related communication (e.g., via FGF19, FGFR4). Our proposed selective MEK inhibitor PD-184352 has a proven ability to suppress FGF-mediated angiogenesis in vivo and decreases VEGF expression [43,44]. A specific pattern in RDEB and OTR was the elevated expression of tyrosine kinase ephrin type A receptor 2 (EPHA2). Emerging evidence links the abundant expression of EPHA2 with high malignant cells. Consequently, in addition to its functional relevance in malignancies, EPHA2 is considered as a liaison to deliver therapeutics to cancer cells [45].

Overall, based on the perturbome data deposited at the LINCS platform, compounds with potential to impact disease-associated de-regulated pathways were identified by extrapolation from SCC-transcriptome data. However, we acknowledge certain limitations in the presented study, which is focused on bioinformatic analysis and the interpretation of transcriptome data. Although we have drawn our conclusions from appropriately large sample sizes supported by the integration of several datasets to provide reliable outcomes, in vitro or in vivo validation is warranted to demonstrate specificity and efficacy, as well as the transferability of the generalized LINCS data to a specific context, such as rare aggressive cutaneous SCCs. However, to date, several publications attest to the innovative application of the LINCS platform, which has facilitated the identification of potential drug candidates for the treatment of various diseases, including cancer [46,47]. Given the tremendous advances in the development of novel in silico screening methods, as well as extensive publicly accessible drug perturbation repositories including FDA/EMA approved compounds during the last years (such as LINCS), there is now ample opportunity to exploit these new exciting tools for accelerated drug development whilst substantially reducing risk and costs. Furthermore, these new approaches in virtual screening also enable non-industrial research labs to conduct drug-screening programs for rare conditions. Future studies are planned to address current limitations and further refine and advance the proposed methodology. 

In summary, we present here a use case describing the implementation of novel computational techniques to advance new cancer treatments for patients with particularly aggressive SCCs. 

## 4. Materials and Methods

### 4.1. Data Sets and Extraction of Differentially Expressed Genes

All data pre-processing, differential expression analysis and generation of uniform manifold approximation and projection for dimension reduction (UMAP) and heatmap plots were performed in the statistical software R [48]. Curated HN-SCC RNA-seq gene expression data were retrieved from “The Cancer Genome Atlas” (TCGA, Bethesda, MD, USA) using the Bioconductor package “curatedTCGAData” [20]. Trimmed mean of M-values method (TMM, calcNormFactors function of “edgeR”) was applied to normalize data. HumanWG-6 v2 Expression BeadChips (Illumina, Munich, Germany) OTR data (GEO repository, Bethesda, MD, USA: GSE32628) was normalized with normalizeBetweenArrays function from the “limma” linear models package [19]. RNA-seq data derived from tissue biopsies (GEO repository: GSE111582) was TMM-normalized and utilized for characterizing the tumor and skin-related transcriptome from patients carrying RDEB-associated mutations in *COL7A1* [14]. In both RNA-seq data sets, genes with low counts were removed by application of the filterByExpr function from the “edgeR” package [49].

Differential gene expression analysis was performed with recommended standard workflows in R packages “limma” for OTR data and “DESeq2” for both HN and RDEB data. Benjamini–Hochberg correction for multiple testing was applied to obtain adjusted *p*-values [50].

### 4.2. Evaluation of Similarities between Data Sets

UMAP was conducted on normalized, log2- and within-data set z-transformed gene expression data (umap function with default parameters from “umap” package) including all genes shared between the three datasets.

The intersection of min. 2-fold de-regulated genes between tumor and control samples with adjusted *p*-value < 0.01 derived from all three tumor entities were defined as “overlapping” DEGs.

Datasets were filtered for overlapping DEGs and respective gene expression data normalized, log2-transformed, centered and scaled (z-transformed within datasets) and represented in a heatmap (pheatmap function). Agglomerative hierarchical clustering of columns and rows was performed with complete linkage on a 1-correlation distance measure.

### 4.3. Gene Set Enrichment Analysis of Overlapping DEGs

Overlapping DEGs were used as input in the Enrichr online enrichment tool. The top ten “Molecular Signature Database” (MsigDB, San Diego, CA, USA) hallmark gene sets that were significantly (adjusted *p*-value < 0.05) enriched were retrieved and represented in barplots, according to significance [51].

### 4.4. Computational Drug Screening

The L1000CDS2 drug perturbation data search engine (maayanlab.cloud/L1000CDS2, New York, NY, USA; 26 November 2021) was used for computational drug screening. Lists of min. 2-fold up-/down-regulated genes from each of the three datasets were used in the query. The search for small molecule signatures was performed on the latest database version in reverse mode. 

### 4.5. Gene Set Enrichment Analysis of Putative Drug Targets

Min. 2-fold up-/down-regulated genes were used in a L1000CDS2 drug search followed by enrichment analysis performed on reported putative targets of the top ranked compounds, with focus on a subset of up-regulated gene patterns, predicted to be reversed by the drug. Top ten significantly (adjusted *p*-value < 0.05) enriched MsigDB hallmark gene sets were retrieved from Enrichr and represented in a barplot, sorted by significance [51]. Gene set enrichment was performed using RDEB data as exemplary. 

### 4.6. MAPK Signaling Pathway Analysis

Log2 foldchange values derived from differential gene expression analysis were mapped to corresponding genes summarized in the KEGG MAPK signaling pathway (hsa04010) using function pathview from R/Bioconductor package Pathview in node.sum = “max” and multi.state = TRUE mode [52]. Genes listed as GFs, RTKs or RAS homologues nodes in the KEGG pathway with significant up-regulation in tumor tissue (min. 2-fold, adj. *p*-value < 0.05) were used for boxplots. Boxplots show normalized count data (RDEB, HN) adjusted with one pseudocount, and signal intensities (OTR), all log2 and z-transformed (centered and scaled) to allow similar scaling between datasets.

## Figures and Tables

**Table 1:** Data sets used for determination of DEG-based tumor signatures.

Data Set	Type	Samples Included	Platform	Reference
RDEB (GSE111582)	tissue	SCC (*n* = 8)	RNA-seq, Illumina HiSeq	[16]
		control (*n* = 9)		
OTR (GSE32979)	tissue	SCC (*n* = 15)	Array, Illumina human-6 v2.0 expression beadchip	[21]
		control (*n* = 13)		
HN (TCGA)	tissue	SCC (*n* = 263)	RNA-seq	[22]
		control (*n* = 31)		

## Data Availability

The data that support the findings of this study are available upon request.
